# Contraceptive Method Utilization and Determinant Factors among Young Women (15-24) in Ethiopia: A Mixed-Effects Multilevel Logistic Regression Analysis of the Performance Monitoring for Action 2018 Household Survey

**DOI:** 10.1155/2021/6642852

**Published:** 2021-04-05

**Authors:** Gurmesa Tura Debelew, Mahilet Berhanu Habte

**Affiliations:** Department of Population and Family Health, Faculty of Public Health, Institute of Health, Jimma University, Jimma, Ethiopia

## Abstract

Despite highly effective modern contraceptive methods (both short and long acting) are made widely available and accessible globally, their utilization remains low among young women in low- and middle-income countries, including Ethiopia. Hence, this study is aimed at determining its status and identifying the determinant factors by using nationally representative data. A multilevel analysis of the nationwide Performance Monitoring for Action 2018 of Ethiopia round 6 data, collected from June to July, 2018, was conducted. A total of 982 both married and unmarried young women (15-24) were included in the analysis. Descriptive statistics was used to describe the status of contraceptive method utilization and unmet need across regions. A mixed-effects multilevel logistic regression model was used to identify the determinants of the contraceptive method utilization. Adjusted odds ratios with corresponding 95% confidence intervals were used to show the significance of the associations at *p* < 0.05. The status of contraceptive method utilization was 54.8% (95% CI: 51.7%, 57.9%), and 18.6% (95% CI: 16.3%, 21.2%) had unmet need. Afar (12.3%), Harari (12.5%), and Ethiopian Somali (20.0%) regions had the lowest contraceptive method utilization. Wealth quintiles and religion were the higher level variables affecting contraceptive method utilization among the young women. Age, marital status, parity, future birth intention, and knowledge of contraceptive methods were the individual level factors identified as determinants of contraceptive method use among the young women. In conclusion, the status of contraceptive method utilization among the young women in Ethiopia is promising as compared to the national target of 55% for 2020; however, still high unmet need exists. The factors also exist both at the contextual and at individual levels. Hence, multilevel interventions need to be in place giving special emphasis to the low performing regions. Besides, region-specific behavioral interventions and family planning services that will be able to reach the young women need to be designed.

## 1. Introduction

Our world is home to more than 1.8 billion young people between the ages of 10 and 24, with more than 600 million adolescent girls having specific needs of youth-friendly health services, including reproductive health and family planning. However, several barriers exist connected in part to their low status in their homes and communities and to their lack of access to the means to decide freely whether, when, or how often to become pregnant. The poor utilization of the existing contraceptive methods has also been a key contributory factor for the high unintended pregnancies and difficulties of fertility regulation among young women. Though age disaggregated data are limited in many countries, the global contraceptive utilization among young women aged 15 to 24 years was 22% in 2014 as compared to 60% among the older women (25 or above) [1].

In the Sub-Saharan Africa (SSA), the utilization of contraceptive methods has been one of the lowest in the world with particular problem among the young women aged 15-24 years. It has also been predicted that avoiding barriers to the use of contraceptive methods and reaching all women with unmet need, including the young women, can prevent the 74 million unintended pregnancies, 25 million unsafe abortions, and 47,000 maternal deaths occurring each year in the low- and middle-income countries [2].

Ethiopia is also one of the countries with high fertility. The Ethiopian Demographic and Health Survey (EDHS) 2016 report has shown that the total fertility rate (TFR) in the country was 4.6 children per woman and the adolescent fertility in the country is one of the highest in the world with teenage pregnancy rate of 12%. The report also has shown that the contraceptive prevalence rate among the young women aged 15-24 was very low (16%) with high unmet need (19%) [3].

The Federal Ministry of Health (FMOH) of Ethiopia had developed a Health Sector Transformation Plan (HSTP) for the years 2016-2020 in 2015, which intended to increase the contraceptive prevalence rate (CPR) from 42% to 55% and reduce the adolescent fertility from 12% to 3% by 2020 [4]. As part of the implementation of the HSTP, the FMOH has also developed Adolescent and Youth Health (AYH) Strategy in 2015 for the years from 2016-2020. The key outcome targets of the AYH strategy were increasing contraceptive prevalence rate to 30% and reducing unmet need for contraception to 10% among young women of 15-24 years by 2020 [5]. To achieve these targets, the country has put strong investments in the health sector in collaboration with different bilateral and multilateral partners to improve the access and utilization of the different contraceptive methods by all women in general and the young women in particular.

However, evidences are limited on assessing the effectiveness of these efforts in increasing the contraceptive method utilization and decreasing the unmet need among women in Ethiopia in general and the young women in particular. The existing local studies that assessed the determinants were more focused on women of reproductive age and limited studies on young women. Besides, they tried to identify factors at individual level without considering factors at different levels, including contextual and individual levels.

Hence, this study is aimed at estimating the status of the contraceptive use and unmet need among the young women aged 15-24 years by using national data and tried to identify the factors at different levels by applying mixed-effects multilevel logistic regression model. Thus, it is hoped that up-to-date evidence on the current status of contraceptive use and unmet need among young women and the determinants at various levels are very much important to deepen the understanding of policy-makers and program planers. The evidence would also direct the development of right strategies and programs so as to close the existing gaps for underutilization of contraceptive methods among young women in Ethiopia. This in turn will enable the country to ensure the achievement of the sustainable development goal 3, which states ensuring universal access to sexual and reproductive health services, including family planning.

## 2. Materials and Methods

### 2.1. Study Setting

This study was conducted in Ethiopia, across the country. Ethiopia is a country situated in Eastern Africa bounded on the North by Eritrea, on the North-East by Djibouti, on the East and South-East by Somalia, on the South by Kenya, and on the West by Sudan. With estimated total population of about 112 million people and annual growth rate of 2.5% in 2019, Ethiopia is the second most populous country in Africa, next to Nigeria, and the 12^th^ most populous country in the world. The country is characterized by predominantly young population with the median age of about 18 years and the young people aged 10-24 accounting for 42% of the total population [5, 6]. Administratively, the country is subdivided into nine regional states and two city administrations at the time of the survey, but now ten regional status and two city administrations.

### 2.2. Data Source

This article was analyzed from the Performance Monitoring for Action (PMA) 2018 of Ethiopia Round 6 (PMA2018/Ethiopia R6) data collected from June to July, 2018. The PMA2018/Ethiopia R6 data collections were facilitated by the Addis Ababa University School of Public Health, Federal Ministry of Health and Central Statistical Agency (CSA) of Ethiopia, in collaboration with the Johns Hopkins University and the Bill and Melinda Gates Institute for Population and Reproductive Health. The PMA Ethiopia is a five-year project, where nationally representative survey measuring key indictors on reproductive, maternal, and newborn health (RMNH), including family planning, by implementing cross-sectional and cohort surveys fill the data gap not currently measured by other large-scale surveys such as EDHS [7]. It started in 2014 (rounds 1 and 2), and then one round every year and the PMA 2018 was round 6. The PMA data are available for researchers for publications on official request from the authorized organization at https://www.pmadata.org/countries/ethiopia.

### 2.3. Study Population

As the aim of this study was to determine the contraceptive method utilization and identify the determinants among the young women, all women aged 15-24 years included in the PMA2018/Ethiopia round 6 survey across the country were considered the study population.

### 2.4. Sample Size Determination

The minimum required sample size for this study was calculated by using EpiInfo statcalc version 3.5.1 considering one population proportion based on the following assumptions. The prevalence of contraceptive use among young women was estimated to be 47.2% from M-EDHS 2019 [8]. The level of confidence of 95%, margin of error of 5%, nonresponse rate of 10%, and a design effect of 2 were also considered. Based on these assumptions, the minimum required sample size became 842. However, as the total number of the eligible young women (15-24) identified during the eligibility assessment from the PMA2018 round 6 was 982, all were included in the analysis as they were close to each other and may also improve the level of precisions.

### 2.5. Sampling Methods

The PMA2018/Ethiopia-Round 6 used a two-stage cluster design with urban-rural, major regions as strata. This survey round 6 used the same 221 enumeration areas (EAs) from the previous rounds drawn by the Central Statistical Agency (CSA) from its master sampling frame. For each EA, 35 households were selected systematically using a random selection. Then, households with eligible females of reproductive age (15-49 years) were contacted and consented for interviews. For the household and female survey from which this data emerged, the final sample included 7,621 households and 7,429 de facto females [7]. For this particular article, during the analysis, all the 982 young women (15-24) eligible for contraceptive method use were identified and included in the study.

### 2.6. Eligibility Assessment

**Figure 1:**
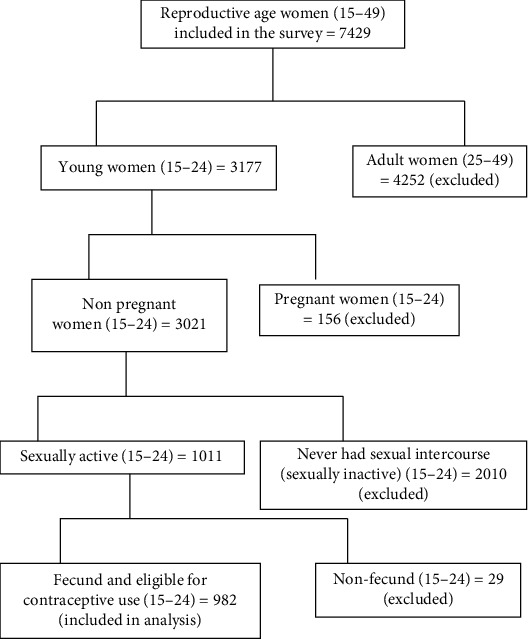
Eligibility assessment of the respondents for contraceptive method utilization among the young women in Ethiopia, June-July, 2018.

To identify edible population for this study, first, young women aged 15-24 years were identified from the 7,429 de facto females included in the survey; missing age was considered noneligible. Second, pregnant women were excluded as they were not eligible for contraceptive method use. Third, sexually active young women were selected by excluding those who never had sexual intercourse. Finally, 982 eligible young women (126 never married and 856 ever married) were identified and included in the analysis after excluding the nonfecund women. The details are indicated in Figure 1.

### 2.7. Data Collection Process

The PMA2018/Ethiopia-Round 6 data were collected by trained data collectors through face-to-face interview of eligible women by using Open Data Kit (ODK). The ODK was helpful in controlling the quality of the data as it displays the list of the geographies entered, enumeration area (EAs), structure number, and household number entered into the household questionnaire linked to the female questionnaire and also uses geographic information system (GPS).

### 2.8. Description of Variables and Measurement

The dependent variable for this study was contraceptive method utilization. Because of the two-stage cluster sampling method, the independent variables were classified into two levels by considering the region as a cluster variable, from now onwards referred as “cluster.” This was because there are some variables that have a clustering effect within the regions and common to some of the respondents and may violate one of the assumptions of the logistic regression that assumes every study subject is independent of each other. In this study for example, when we look at the religion, Afar, Somali, and Harari regions are Muslim dominant and Amhara and Tigray regions are Orthodox Christian dominant, while the others are mixed type. Similarly, some regions (Addis Ababa, Dire Dawa, and Harari) are completely urban and the rest have both rural and urban residents.

To avoid the violation of the assumption of the independence, the independent variables were categorized into two levels: level 2 (cluster level variables) also called higher level variables (place of residence, religion, and wealth quintiles) and level 1 (individual level variables) also called lower level variables. The lower (individual level) variables included age, educational status, marital status, family size, parity, intention when to have the next birth, and knowledge on contraceptive methods. The detailed description of the variables and measurements are indicated in Table 1.

### 2.9. Data Management and Analysis

After careful cleaning and exploring of the data, descriptive statistics were performed by determining the frequency distributions and percentages along the different demographic and reproductive characteristics as well as contraceptive method utilization. Then, bivariate analysis was done by cross-tabulating each independent variable with contraceptive method utilization to identify variables having significant association at *p* < 0.05.

Due to the multistage cluster sampling, individual women living in the same region (cluster) were expected to share similar characteristics (communalities) than those from other regions. Hence, the assumption of independence among women within the same cluster and that of equal variance across clusters are not valid. Considering this hierarchical nature of the data, women were nested within the regions. Women living in the same region (cluster) may share similar characteristics like religious believes, access to care, and socioeconomy. Thus, the estimates from the ordinary logistic regression that assumes all individual women who are independent would not be valid. Therefore, by considering this hierarchical structure of the data, women were nested within the regions, so that the model enables partitioning of the total variation in the outcome into within-group (within region) and between-group (between regions) so as to differentiate the relative contributions of individual women-related variables and the common variables within the region. Because of this, a two-level mixed effects multilevel logistic regression model with a random intercept was performed by using Stata 13 to identify the determinants of contraceptive method utilization.

To evaluate the significance of the region-level clustering of the dependent variables (random effects), the intraclass correlation coefficient (ICC) was calculated in the empty model. The association between independent and dependent variables was estimated by using odds ratios at 95% confidence intervals in the final full model. The detailed results of the model evaluation are indicated under the result section.

## 3. Results

### 3.1. Demographic and Reproductive Characteristics of the Respondents

Among the 982 young women aged 15-24 included in the analysis, nearly three-quarter (724 (73.7%)) were in the age range of 20-24. Slightly more than half (512 (52.4%)) were from rural residents. About one in five (188 (19.1%)) never attended formal education and 452 (46%) only attended primary education. The great majority (807 (82.2%)) of the respondents were either married or in union. About half (487 (49.6%)) were Orthodox Christians, and nearly two in five were in the highest wealth quintile. Half (494 (50.4%)) had family size of 3-4 people; 560 (57.0%) had 1-2 births, and about three-quarter (736 (74.9%)) had the intention to have the next birth after 2 years (Table 2).

### 3.2. Knowledge of the Respondents on Contraceptive Methods

**Figure 2:**
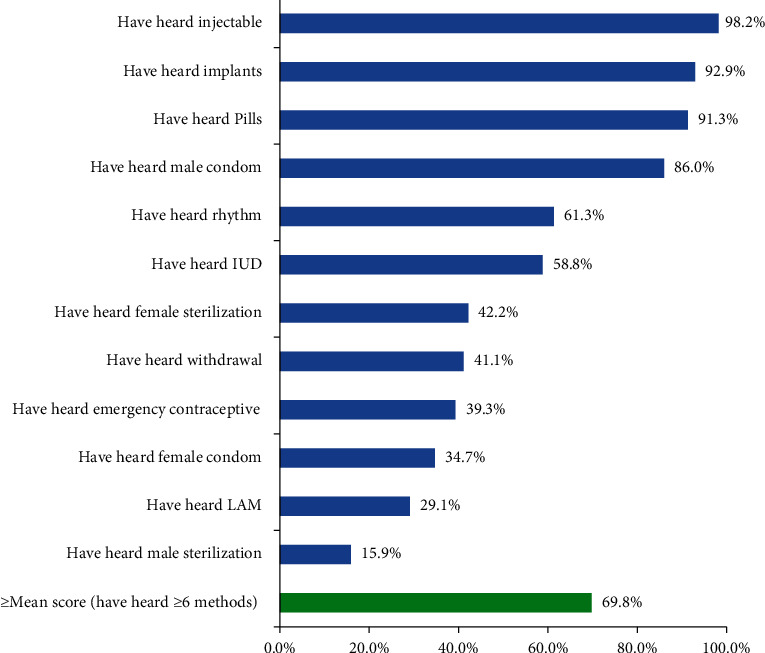
Knowledge of contraceptive methods among young women in Ethiopia, June-July, 2018.

As indicated in Figure 2, the respondents were asked whether they had heard any of the 12 contraceptive methods. Then, a composite index was produced by adding the 12 responses and a mean score was determined, and it was 6. Accordingly, 685 (69.8%) of the respondents had heard 6 or more of the 12 contraceptive methods (greater or equal to the mean score) and labeled as having good knowledge. Injectables (964 (98.2%)), implants (912 (92.9%)), and pills (897 (91.3%)) were the most commonly mentioned contraceptive methods by the respondents, whereas male sterilization (156 (15.9%)), Lactational Amenorrhea Method (LAM) (286 (29.1%)), and female sterilization (341 (34.7%)) were the least known contraceptive methods by the respondents (Figure 2).

### 3.3. Contraceptive Method Utilization

**Figure 3:**
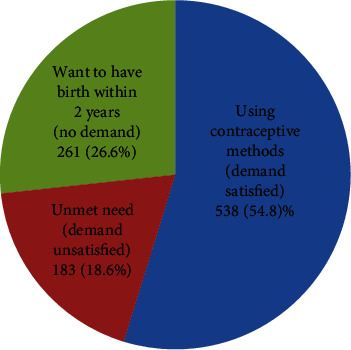
Contraceptive method utilization and unmet need among young women in Ethiopia, June-July, 2018.

Among the 982 young women included in the analysis, 538 (54.8%; 95% CI: 51.7%, 57.9%) were using any contraceptive method (their demand satisfied) during the time of the survey (current users). As they were young women, all, except 2 respondents, were used for spacing. On the other hand, about one-in-five, 183 (18.6%; 95% CI: 16.3%, 21.2%) were not using any contraceptive method while they had a need, unmet need (demand unsatisfied). This makes the total demand for contraceptive methods to be 721 (73.4%; 95% CI: 70.5%, 76.2%) [Figure 3].

### 3.4. Regional Variations in Contraceptive Method Utilization

**Figure 4:**
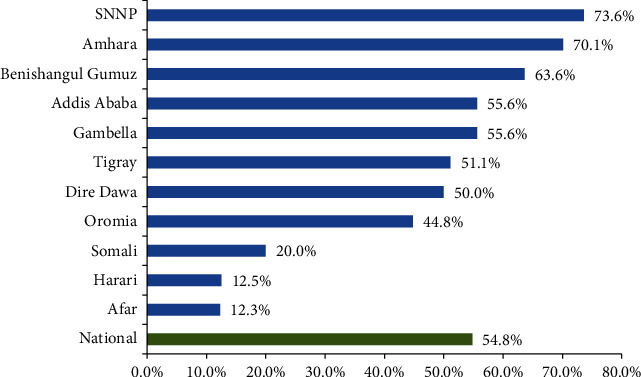
Regional variations in contraceptive method utilization among young women in Ethiopia, June-July, 2018.

Regarding the regional variation of the contraceptive method utilization, Afar (12.3%), Harari (12.5%), and Ethiopian Somali (20.0%) were found to be the three regions with the lowest utilization rate, whereas South Nations Nationalities and People (SNNP) (73.6%), Amhara (70.1%), and Benishangul-Gumuz (63.6%) regions were found to have the highest contraceptive utilization rate by the young women [Figure 4].

### 3.5. Contraceptive Method Mix

**Figure 5:**
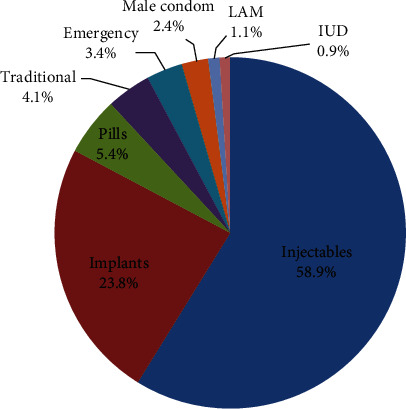
Contraceptive methods mixed by young women in Ethiopia, June-July, 2018.

Among the 982 respondents, most (317 (58.9%)) were using injectables, whereas the long acting reversible contraceptive (LARC) methods (implants and IUD) were used by only a quarter of the respondents (133 (24.7%)). Moreover, still 4.1% rely on traditional methods (Figure 5).

### 3.6. Regional Variations in Unmet Need for Contraceptive Methods

**Figure 6:**
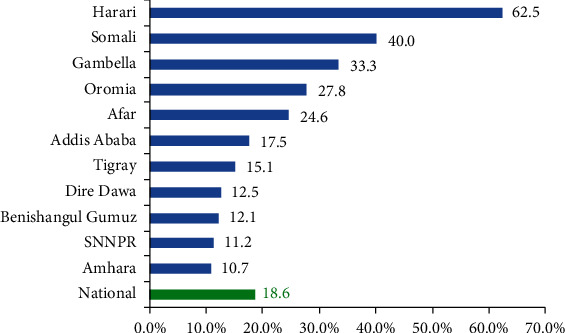
Regional variations in unmet need for contraceptive methods among young women in Ethiopia, June-July, 2018.

As can be seen from Figure 6, the status of unmet need at the national level is 18.6%. Harari (62.5%), Somali (40.0%), and Gambela (33.3%) regions had the highest unmet need for contraceptive methods [Figure 6].

### 3.7. Determinants of Contraceptive Method Utilization

#### 3.7.1. Bivariate Analysis

In the initial bivariate analysis, all the three cluster-level variables (place of residence, wealth quintiles, and religion) had statistically significant association with contraceptive method utilization at *p* < 0.05. Among the individual level variables, age, educational status, marital status, family size, parity, future pregnancy intention, and knowledge of contraceptive methods had statistically significant association with contraceptive method utilization at *p* < 0.05.

#### 3.7.2. Model Applicability Evaluation

To evaluate the applicability of the two-level mixed effects logistic regression model, the intraclass correlation coefficient (ICC(*ρ*)) was calculated in the empty model (Model 0) and it was found to be 0.579 indicating that 57.9% of the variation was contributed by between-cluster variation. The result of the test of the significance of the log likelihood (LR) vs. logistic regression showed strongly significant variation across clusters (regions) (*p* < 0.0001). This suggested that adding between-cluster variance in the model was necessary to detect the effect of each variable on the contraceptive method utilization by the young women.

To further evaluate the applicability of the mixed effects-model, additional three models were run. In Model 1, all the three cluster-level variables were included and the ICC(*ρ*) became 0.580 (58.0%) and the test of the preference of the log likelihood (LR) vs. logistic regression was significant (*p* < 0.0001). In Model 2, all the individual level variables were included and the ICC(*ρ*) became 0.575 (57.5%) and the test of the preference of the log likelihood (LR) vs. logistic regression was again significant (*p* < 0.0001). Finally, the full model (Model 3) was run by including all the cluster level and individual level variables and the ICC(*ρ*) was 0.578 (57.8%) and the test of the preference of the log likelihood (LR) vs. logistic regression was again strongly significant (*p* < 0.0001). Hence, it was decided that the use of mixed-effects two-level logistic regression model is preferred as compared to the ordinary logistic regression [Table 3].

#### 3.7.3. Mixed-Effects Multilevel Logistic Regression Analysis

After adjusting in the final mixed-effects two-level logistic regression model (Model 3), among the cluster-level variables, wealth quintiles and religion had statistically significant association with contraceptive method utilization by the young women. Among the individual level variables, age, marital status, parity, future birth intention, and knowledge on contraceptive methods had statistically significant association with contraceptive method utilization at *p* < 0.05.

Young women in the higher (AOR = 2.00; 95% CI: 1.08, 3.72) and highest (AOR = 2.83; 95% CI: 1.35, 5.95) wealth quintiles were more likely to utilize contraceptive methods as compared to those in the lowest quintile. Among the different religious categories, young women who belong to Muslim were about 50% less likely to utilize the contraceptive methods as compared to the Orthodox Christian followers (AOR = 0.50; 95% CI: 0.33, 0.76). Young women who were in the age group of 20-24 were more likely to utilize contraceptive methods as compared to those in the age group of 15-19 years (AOR = 1.66; 95% CI: 1.14, 2.43).

Young women who were not married (AOR = 1.61; 95% CI: 1.01, 2.81) and those who were divorced (AOR = 3.20; 95% CI: 1.30, 7.09) were more likely to utilize contraceptive methods as compared with those who were married or in union. Similarly, young women who had 1-2 births (AOR = 0.51; 95% CI: 0.30, 0.86) and who had three or more births (AOR = 0.38, 95% CI: 0.17, 0.87) were less likely to utilize the contraceptive methods as compared with those who had no birth at all (nulliparous). Young women who had future intention to have the next birth after two years (AOR = 11.38; 95% CI: 6.46, 20.04) and those who were not decided when to have (AOR = 8.81; 95% CI: 4.32, 17.96) were more likely to utilize the contraceptive methods as compared to those who had a plan to have soon within 2 years. Young women who had relatively good knowledge on contraceptive methods were more than two times more likely to utilize as compared to those who had poor knowledge (AOR = 2.15; 95% CI: 1.47, 3.16) [Table 4].

## 4. Discussion

This study tried to determine the status of contraceptive method utilization and identify the determinant factors among young women in Ethiopia. Accordingly, 54.8% of the respondents were using any method of contraception during the time of the survey. This finding is similar with the 55% outcome target set in the health sector transformation plan (HSTP) and the adolescent and youth health (AYH) strategy of the country, Ethiopia, to be attained by 2020 [9, 10]. But this finding is slightly higher than the finding of the 2019 Ethiopian Mini-Demographic and Health Survey (M-EDHS-2019) report (47.2%; 36.5% among 15-19 and 52.5% among 20-24) [8]. This difference may be due to the deference in the denominator or exclusion criteria. In this study, pregnant and nonfecund women were excluded from the analysis, while not excluded in the M-EDHS. Had this been included in the current analysis, the prevalence of contraceptive use would be 46.1%, but underestimated because of the inclusion of those not at risk of pregnancy.

In this study, Afar (12.3%), Harari (12.5%), and Ethiopian Somali (20.0%) regions were found to have the lowest coverage of contraceptive method utilization among young women. This is also in line with the findings of M-EDHS 2019, in which the contractive prevalence rate among the married reproductive age women in Ethiopian Somali (3.4%), Afar (12.7%), and Harari (32.4%) was low as compared to other regions [8]. Though this may be partly due to cultural and religious backgrounds that encourage high fertility in these regions, further in-depth investigation of programmatic issues and intervention-related barriers is required.

The Ethiopian National Health Sector Transformation Plan (2016-2020) and the adolescent and youth health strategy of 2016-2020 have set a target of increasing the long acting reversible contraceptive (LARC) methods to be at least 50% of the methods mix [4, 5]. But in this study, LARC methods (implants and IUD) accounted only half of this target (24.7%), whereas using injectables (short-acting) was the commonly utilized method accounting for 58.9% of the methods mix and 4.1% still relied on traditional methods. Similarly, in the M-EDHS 2019, the LARC methods accounted for 11% of the 41% contraceptive prevalence rate (CPR) (26.8% of the methods mix) and injectables accounted for 27% of the 41% CPR (65.9% of the method mix). This low coverage of LARC methods and higher preference of injectables have also been reported in previous studies conducted in other parts of the country among the young women as well as the reproductive age women [9–11]. This may be due to the more familiarity of the young women to the short-term methods as well misconceptions or perceived side effect of the long acting as causing delayed fertility to the young women.

In this study, the determinants of contraceptive method utilization by young women existed at both cluster and individual levels. Among the cluster level variables, religion was found to affect the contraceptive use among the young women, where Muslim women were about 50% lower as compared to Orthodox Christians. This also supports the above regional variations and the clustering effect of religion as 75% of the respondents in Harari, 87.7% in Afar and 100% in Ethiopian Somali in the study belong to Muslim in religion. This finding has been reported in previous studies conducted in the country [12, 13]. This also has important programmatic implication of further investigation and adequate engagement of religious leaders in the promotion of contraceptive method utilization by the young women in these regions.

In this study, young women who belong to the higher and highest wealth quintiles were more likely to utilize the contraceptive methods as compared to the lowest quintile. This finding has also been reported in previous studied conducted in other African countries [14, 15]. This may be due to the fact that young women with a better socioeconomic status may have better access to services and better care for their health while the poor women in low income settings focus on daily activities for their families' survival than their own health care. This also has an important programmatic implication of special emphasis to young women with low socioeconomic status while designing family planning programs, including them at home with the service.

Women in the age group of 20-24 years were more likely to use contraceptive methods as compared to the adolescent age group (aged 15-19). This is also consistent with previous studies conducted in Ethiopia as well as other countries in Africa that teenagers were less likely to use contraceptive methods as compared to older women [9–11, 16]. This may be due to lack of adolescent friendly sexual and reproductive health services in most parts of the country.

In this study, young women who were married or in union were less likely to use the contraceptive methods as compared to those who were not married or divorced. This finding has also been reported in studies conducted in Northwest Ethiopia [10], Southern Ethiopia [11], Ghana [15], Tanzania [16] and a study conducted in 73 low- and middle-income countries [17]. This might be due to the reason that young women in a marital union are more likely to have a desire to have child and the unmarried or divorced once wanted to avoid unwanted pregnancy out of marriage and more likely to use contraceptive methods.

Theoretically, it is assumed that those women having a large number of children (multiparous) are more likely to use contraceptive methods as compared to the nulliparous women. However in this study, in the contrary, as parity increases, the contraceptive method use decreases. Similar findings have been reported in studies conducted in Southern Ethiopia [11], Oromia Region Ethiopia [19], and Ghana [15] and the study conducted among 73 low- and middle-income countries [17]. The possible explanation could be the nulliparous women were not having birth as they were using contraceptive methods and they may be more likely those who were not married.

Similarly, those who have the intention to have birth after two years or those who have not decided yet when to have the next birth were more likely to use contraceptive methods as compared to those who have the intention to have after two years. This finding is in line with the findings of previous studies conducted in Ethiopia as well as other African countries where future birth intention was one of the predictors of contraceptive method use [11, 15, 18].

Having good knowledge on the available contraceptive methods was also found to increase the contraceptive use more than two-fold in this study. This finding has also been reported in previous studies conducted in Gondar, Ethiopia [9], Southern, Ethiopia [11], and Tanzania [16]. This has a programmatic implication of strengthening the behavioral change interventions at the facility as well as at the household levels.

Place of residence and educational level of the women had significant association with the contraceptive use in previous studies conducted in Ethiopia and abroad [9, 15–18]. However, in this study, the two variables had nonsignificant association in the mixed-effect multilevel analysis. This implies that among the young women, being in urban residence or having better education alone may not increase contraceptive method utilization unless there are additional behavioral interventions to increase the level of knowledge of the young women on contraceptive methods.

This study may have its own limitations in that factors related to supply side and husband related factors were not addressed in the PMA survey.

## 5. Conclusion

The status of contraceptive method utilization among the young women in Ethiopia is promising as compared to the set target of 55% for the year 2020. However, the unmet need for contraceptives of 18.6% is higher than the national target of 10% for 2020. Harari, Somali, and Gambela regions had the highest unmet need. A clustering effect of religion and low utilization of the contraceptive methods were detected in Afar, Harari, and Ethiopian Somali regions. Socioeconomy, age, marital status, parity, future birth intention, and knowledge of contraceptive methods were identified as determinants of contraceptive method use among young women in Ethiopia.

Hence, designing context-specific interventions and engaging the religious leaders; strengthening region-specific behavioral interventions to raise the level of knowledge, including strategies to reach the unmarried and divorced women; and improving access to contraceptive methods in the regions with low coverage and high unmet need are recommended.

## Figures and Tables

**Table 1:** Description of the variables and measurements for the multilevel logistic regression analysis of the determinants of contraceptive method utilization among young women in Ethiopia, June-July, 2018.

Variables	Descriptions	Measurements
*Dependent variable*		
Contraceptive method utilization	Using any contraceptive methods (modern or traditional) during the time of the survey	A woman who was using any one of the contraceptive methods was coded as “1” and who was not using as “0”

*Level 2 (higher level) independent variables*	*Communal (cluster level) variables*	
Place of residence	The usual place of residence where the woman lives	Urban kebele was coded as “1,” and rural kebele was coded as “0”
Wealth quintiles	House hold assets, home, land, and livestock's ownership were assessed, and wealth index was computed by using principal component analysis (PCA)	The wealth status was categorized into five groups and ranked from the poorest to the wealthiest quintile
Religion	The religious background of the respondent	Each religion was entered and later recoded as “Orthodox Christian,” “Muslim,” “Protestant,” and “Others.” Others were merged because they were very few for logistic regressions

*Level-1 (lower-level) independent variables*	*Individual level variables*	
Age group	Age of women at interview in completed years	Asked in completed years and later categorized into “15-19” and “20-24”
Educational status	Highest level of education attained by the respondent	Categorized into 4 groups as “No formal education,” “Primary (1–8),” “Secondary (9–12),” and “Tertiary (12+)”
Marital status	Marital status of the woman at the time of interview	Categorized into 3 groups as “Never married,” “Married or in union,” and “Divorced”
Family size	Number of people living in the same room with a respondent as a family member	Recorded in absolute number and later codded as “1-2,” “3-4,” and “≥5”
Parity	Number of births the woman ever had	Collected in absolute number and latter categorized into “Nulipara,” “1-2 births,” and “≥3 births”
Intended next birth	Intended or planed time to have the next birth	Categorized as “Within 2 years,” “After 2 years,” and “Not decided”
Knowledge on contraceptive methods	The women were asked whether they have heard each of the 12 methods of contraceptive methods, and a composite knowledge score was produced by using PCA	Mean score was computed for the composite knowledge score; those scored above or equal to the mean score were categorized into having “Good knowledge,” and those who scored less than the mean were categorized as “Poor knowledge”

**Table 2:** Demographic and reproductive characteristics of the respondents, Ethiopia, June-July, 2018 (*n* = 982).

Variables	Categories	Frequency	Percentage
Age group (in years)	15-19	258	26.3
	20-24	724	73.7

Place of residence	Rural	515	52.4
	Urban	467	47.6

Educational status	No formal education	188	19.1
	Primary (grade 1-8)	452	46.0
	Secondary (grade 9-12)	218	22.2
	Technic/college (12+)	124	12.6

Marital status	Never married	126	12.8
	Married or in union	807	82.2
	Divorced	49	5.0

Religion	Orthodox Christian	487	49.6
	Muslim	328	33.4
	Protestant	155	15.8
	Others^∗^	12	1.2

Wealth quintiles	Lowest quintile	124	12.6
	Lower quintile	117	11.9
	Middle quintile	135	13.7
	Higher quintile	232	23.6
	Highest quintile	374	38.1

Family size	1-2	244	24.9
	3-4	495	50.4
	≥5	243	24.7

Parity	Nulipara (no birth)	359	36.6
	1-2 births	560	57.0
	≥3 births	63	6.4

Intended next birth	Soon	136	13.9
	After 2 years	736	74.9
	Not decided	110	11.2

^∗^Catholic, Jova, and Traditional.

**Table 3:** Test of goodness-of-fit of the mixed-effects multilevel logistic regression model to identify the determinants of contraceptive method utilization among young women in Ethiopia, June-July, 2018.

Models	Random effects as level 2 variance (var(-cons))	Intraclass correlation coefficient (ICC(*ρ*))	Log likelihood (LR) deviance	Wald *χ*^2^	Significance of LR test vs. logistic regression (*p* value)
Model 0	4.53	0.579 = 57.9%	646.28	Reference	<0.0001
Model 1	4.55	0.580 = 58.0%	616.20	56.67	<0.0001
Model 2	4.46	0.575 = 57.5%	568.41	119.08	<0.0001
Model 3	4.52	0.578 = 57.8%	555.65	135.54	<0.0001

(i) Dependent variable: contraceptive method utilization; (ii) cluster variable: region (11 in number); (iii) Model 0: empty model; Model 1: cluster level variables included; Model 2: individual level variables included; Model 3: full model (all the cluster level and individual level variables included.

**Table 4:** Mixed-effects multilevel logistic regression analysis of the determinants of contraceptive method utilization among young women in Ethiopia, June-July, 2018.

Variables	Contraceptive use		Crude OR (95% CI)	Model 1 (level 2 variables) AOR (95% CI)	Model 2 (level 1 variables) AOR (95% CI)	Model 3 (full model) AOR (95% CI)
	Yes (538) *n* (%)	No (444) *n* (%)				
*Level 2: higher (contextual) level variables*						
Place of residence						
Rural	237 (46.0)	166 (54.0)	1.00	1.00		1.00
Urban	301 (64.5)	166 (35.5)	2.13 (1.64, 2.78)	0.86 (0.53, 1.39)		0.90 (0.53, 1.52)
Wealth quintiles						
Lowest quintile	48 (38.7)	76 (61.3)	1.00	1.00		1.00
Lower quintile	45 (38.5)	72 (61.5)	0.99 (0.59, 1.66)	1.40 (0.78, 2.53)		1.41 (0.75, 2.68)
Middle quintile	66 (48.9)	69 (51.1)	1.51 (0.92, 2.48)	1.69 (0.95, 2.98)		1.59 (0.86, 2.95)
Higher quintile	134 (57.8)	98 (42.2)	2.17 (1.39, 3.38)	2.50 (1.42, 4.37)		2.00 (1.08, 3.72)
Highest quintile	245 (65.5)	129 (34.5)	3.01 (1.98, 4.58)	3.82 (1.97, 7.41)		2.83 (1.35, 5.95)
Religion						
Orthodox Christian	310 (63.7)	177 (36.3)	1.00	1.00		1.00
Muslim	118 (36.0)	210 (64.0)	0.32 (0.24, 0.43)	0.42 (0.29, 0.62)		0.50 (0.33, 0.76)
Protestant	104 (67.1)	51 (32.9)	1.16 (0.79, 1.71)	1.05 (0.64, 1.71)		1.22 (0.72, 2.07)
Others^∗^	6 (50.0)	6 (50.0)	0.57 (0.18, 1.80)	0.60 (0.17, 2.08)		0.67 (0.17, 2.67)
*Level 1: lower (individual) level variables*						
Age group (in years)						
15-19	127 (49.2)	131 (50.8)	1.00		1.00	1.00
20-24	411 (56.8)	313 (43.2)	1.35 (1.02, 1.80)		1.65 (1.14, 2.39)	1.66 (1.14, 2.43)
Educational status						
No formal education	62 (33.0)	126 (67.0)	1.00		1.00	1.00
Primary (grade 1-8)	249 (55.1)	203 (44.9)	2.49 (1.75, 3.56)		1.49 (0.95, 2.34)	1.19 (0.74, 1.92)
Secondary (grade 9-12)	147 (67.4)	71 (32.6)	4.21 (2.78, 6.38)		1.76 (1.01, 3.08)	1.16 (0.63, 2.14)
Technic/college (12+)	80 (64.5)	4 4(35.5)	3.40 (2.29, 5.96)		1.08 (0.57, 2.05)	1.67 (0.68, 2.34)
Marital status						
Never married	83 (65.9)	43 (34.1)	1.78 (1.20, 2.64)		2.37 (1.37, 3.80)	1.61 (1.01, 2.81)
Married or in union	420 (52.0)	387 (48.0)	1.00		1.00	1.00
Divorced	35 (71.4)	14 (28.6)	2.31 (1.22, 4.35)		3.05 (1.25, 7.48)	3.20 (1.30, 7.09)
Family size						
1-2	154 (63.1)	90 (36.9)	1.00		1.00	1.00
3-4	271 (54.7)	224 (45.3)	0.71 (0.52, 0.97)		0.93 (0.57, 1.54)	1.05 (0.63, 1.76)
≥5	113 (46.5)	130 (53.5)	0.51 (0.35, 0.73)		0.55 (0.33, 0.91)	0.70 (0.42, 1.18)
Parity						
Nulipara (no birth)	213 (59.3)	146 (40.7)	1.00		1.00	1.00
1-2 births	306 (54.6)	254 (45.4)	0.83 (0.63, 0.99)		0.55 (0.33, 0.91)	0.51 (0.30, 0.86)
≥3 births	19 (30.2)	44 (69.8)	0.30 (0.17, 0.53)		0.38 (0.17, 0.85)	0.38 (0.17, 0.87)
Intended next birth						
Soon	24 (17.6)	112 (82.4)	1.00		1.00	1.00
After 2 years	458 (62.2)	278 (37.8)	7.69 (4.83, 12.24)		10.49 (6.02, 18.29)	11.38 (6.46, 20.04)
Not decided	56 (50.9)	54 (49.1)	4.84 (2.72, 8.63)		8.26 (4.12, 16.55)	8.81 (4.32, 17.96)
Knowledge on contraceptive methods						
Poor knowledge	106 (35.7)	191 (64.3)	1.00		1.00	1.00
Good knowledge	432 (63.1)	253 (36.9)	3.08 (2.32, 4.09)		2.49 (1.73, 3.60)	2.15 (1.47, 3.16)

^∗^Catholic, Jova, and Traditional.

## Data Availability

All related data used to support the findings of this study are included within the manuscript, and the SPSS data set is available from the author and can be obtained on request.

## References

[1] United Nations Population Fund (UNFPA) *State of world population 2014. The power of 1.8 billion: Adolescents, youth and the transformation of the future*.

[2] World Health Organization (WHO) *High rates of unintended pregnancies linked to gaps in family planning services: New WHO study*.

[3] Central Statistics Agency (CSA) [Ethiopia], ICF *Ethiopian Demographic and Health Survey 2016. Addis Ababa, Ethiopia, and Rockville*.

[4] Federal Democratic Republic of Ethiopia-Ministry of Health (FDRE-MOH) *Health Sector Transformation Plan (HSTP) 2016-2020*.

[5] Federal Democratic Republic of Ethiopia-Ministry of Health (FDRE-MOH) *National Adolescent and Youth Health Strategy (2016-2020)*.

[6] United Nations (UN) *Department of Economic and Social Affairs Population Division, World Population Prospects 2019*.

[7] Addis Ababa University School of Public Health and The Bill & Melinda Gates Institute for Population and Reproductive Health at The Johns Hopkins Bloomberg School of Public Health (2018). *Performance Monitoring for Action Ethiopia (PMA-ET) Household and Female Cross-sectional Survey 2018*.

[8] Ethiopian Public Health Institute (EPHI) and ICF *Ethiopia Mini Demographic and Health Survey 2019: Key Indicators*.

[9] Oumer M., Manaye A., Mengistu Z. (2020). Modern contraceptive Method utilization and associated factors among women of reproductive age in Gondar City, Northwest Ethiopia. *Open Access Journal of Contraception*.

[10] Megabiaw B. (2012). Awareness and utilization of modern contraceptives among street women in North-West Ethiopia. *BMC Women's Health*.

[11] Endriyas M., Eshete A., Mekonnen E., Misganaw T., Shiferaw M. N., Ayele S. (2017). Contraceptive utilization and associated factors among women of reproductive age group in Southern Nations Nationalities and Peoples’ Region, Ethiopia: cross-sectional survey, mixed-methods. *Contraception and Reproductive Medicine*.

[12] Tigabu S., Demelew T., Seid A., Sime B., Manyazewal T. (2018). Socioeconomic and religious differentials in contraceptive uptake in Western Ethiopia: a mixed-methods phenomenological study. *BMC Women's Health*.

[13] Worku A. G., Tessema G. A., Zeleke A. A. (2015). Trends of modern contraceptive use among young married women based on the 2000, 2005, and 2011 Ethiopian Demographic and Health Surveys: a multivariate decomposition analysis. *PLoS One*.

[14] Adebowale S., Adedini S., Ibisomi L., Palamuleni M. (2014). Differential effect of wealth quintile on modern contraceptive use and fertility: evidence from Malawian women. *BMC Women's Health*.

[15] Achana F., Bawah A., Jackson E. (2015). Spatial and socio-demographic determinants of contraceptive use in the Upper East region of Ghana. *Reproductive Health*.

[16] Nsanya M., Atchison C., Bottomley C., Doyle A., Kapiga S. (2019). Modern contraceptive use among sexually active women aged 15–19 years in North-Western Tanzania: results from the Adolescent 360 (A360) baseline survey. *BMJ Open*.

[17] de Vargas Nunes Coll C., Ewerling F., Hellwig F., de Barros A. J. D. (2019). Contraception in adolescence: the influence of parity and marital status on contraceptive use in 73 low-and middle income countries. *Reproductive Health*.

[18] O’Regan A., Thompson G. (2017). Indicators of young women’s modern contraceptive use in Burkina Faso and Mali from demographic and health survey data. *Contraception and Reproductive Medicine*.

[19] Kebede A., Abaya S. G., Merdassa E., Bekuma T. T. (2019). Factors affecting demand for modern contraceptives among currently married reproductive age women in rural Kebeles of Nunu Kumba district, Oromia, Ethiopia. *Contraception and Reproductive Medicine*.

